# Examining a Fully Automated Mobile-Based Behavioral Activation Intervention in Depression: Randomized Controlled Trial

**DOI:** 10.2196/54252

**Published:** 2024-08-30

**Authors:** Nicholas Santopetro, Danielle Jones, Andrew Garron, Alexandria Meyer, Keanan Joyner, Greg Hajcak

**Affiliations:** 1 Department of Psychology, Florida State University, Tallahassee, FL, United States; 2 School of Education and Counseling Psychology, Santa Clara University, Santa Clara, CA, United States; 3 Department of Psychology, University of California, Berkeley, Berkeley, CA, United States

**Keywords:** digital intervention, digital health, digital application, digital applications, mobile health, mHealth, automation, automate, automated, behavioral activation, BA, BA intervention, depression, depressed, depressive, depressive symptoms, anhedonia, anhedonia symptoms, anxiety, anxious, anxiety symptoms, adults, adult, psychiatry, psych, psychology, major depressive disorder, MDD

## Abstract

**Background:**

Despite significant progress in our understanding of depression, prevalence rates have substantially increased in recent years. Thus, there is an imperative need for more cost-effective and scalable mental health treatment options, including digital interventions that minimize therapist burden.

**Objective:**

This study focuses on a fully automated digital implementation of behavioral activation (BA)—a core behavioral component of cognitive behavioral therapy for depression. We examine the efficacy of a 1-month fully automated SMS text message–based BA intervention for reducing depressive symptoms and anhedonia.

**Methods:**

To this end, adults reporting at least moderate current depressive symptoms (8-item Patient Health Questionnaire score ≥10) were recruited online across the United States and randomized to one of three conditions: enjoyable activities (ie, BA), healthy activities (ie, an active control condition), and passive control (ie, no contact). Participants randomized to enjoyable and healthy activities received daily SMS text messages prompting them to complete 2 activities per day; participants also provided a daily report on the number and enjoyment of activities completed the prior day.

**Results:**

A total of 126 adults (mean age 32.46, SD 7.41 years) with current moderate depressive symptoms (mean score 16.53, SD 3.90) were recruited. Participants in the enjoyable activities condition (BA; n=39) experienced significantly greater reductions in depressive symptoms compared to participants in the passive condition (n=46). Participants in both active conditions—enjoyable activities and healthy activities (n=41)—reported reduced symptoms of anxiety compared to those in the control condition.

**Conclusions:**

These findings provide preliminary evidence regarding the efficacy of a fully automated digital BA intervention for depression and anxiety symptoms. Moreover, reminders to complete healthy activities may be a promising intervention for reducing anxiety symptoms.

## Introduction

Depressive disorders remain one of the most prevalent and burdensome mental health disorders worldwide [1-3]. Individuals with depression are more at risk for suicide and substance abuse, experience significant occupational impairments (eg, missing work), and have reduced life expectancy [4,5]. Research has also highlighted that anhedonia, a core depressive symptom characterized by a reduced ability to experience pleasure or enjoyment, is particularly pernicious as it is linked to worse treatment responsiveness and depression remission [6,7].

Despite advancements in our understanding and identification of depression, prevalence rates of depressive disorders have generally remained constant in the general population over the past decades [8,9]. However, recent studies examining the impact of the COVID-19 pandemic on internalizing disorders suggest substantial increases in rates of depressive disorders [10-12]. Although evidence-based treatments such as cognitive behavioral therapy (CBT) and behavioral activation (BA) have been extensively underscored as effective psychotherapy interventions for depression, current shortages of professionals in the United States mental health care system are significantly compounding the burden of these rising depression rates. More specifically, in the United States in 2018, approximately 115 million people reported living in a mental health professional shortage area, and approximately 25% of adults with a current mental health disorder did not receive treatment [13]. Thus, depressive disorders remain a persistent and taxing global public health issue, and there is a crucial need for briefer, more accessible, and more affordable evidence-based mental health interventions for depression to supplement these shortages in mental health services.

Digital mental health interventions are one possible solution for making evidence-based psychotherapy treatments more accessible and less expensive. Digital interventions involve using digital technology, such as smart devices, the internet, or mobile apps, to foster or support behavior change. For example, more recent research efforts have examined the effectiveness of chatbot-based interventions in increasing positive affect [14]. Research examining the effectiveness of digital interventions for the treatment of mental health problems has increased rapidly over the past three decades: recent reviews and meta-analyses suggest that digital interventions appear to be effective at reducing symptoms of depression [15-17]. There was a medium overall effect size of digital interventions for depression when compared to control conditions (Hedges g=0.52), and the vast majority (74%) of digital interventions used to treat depressive disorders rely on the tenets of CBT as their theoretical foundation [16]. Importantly, this meta-analysis did not find differences in treatment effect sizes between digital interventions and more traditional in-person psychotherapy for depression.

Various meta-analytic reviews have concluded that BA, a specific aspect of CBT for depression, is itself a highly effective treatment for depressive disorders [18,19]. From the perspective of BA, depressive symptoms are a result of few positive experiences coincident with increases in negative experiences, which then lead to a diminished pursuit for further positive experiences [20]. BA treatment therefore attempts to disrupt this negative feedback loop by purposefully increasing an individual’s participation in enjoyable or rewarding (ie, positive) activities to increase positive mood and reduce negative affect [21].

Past studies have examined the effectiveness of digital BA interventions for depression, and results indicate that in nonclinical settings, guided internet-based BA interventions were equivalent to other forms of behavioral therapy and superior to waitlist conditions at reducing depression and anxiety outcomes [22]. Despite these promising findings, existing digital BA interventions still require a relatively high level of therapist guidance or interaction from the study team. For example, Ly and colleagues [23] administered an 8-week smartphone-based BA intervention that required a trained therapist to contact each participant weekly for no longer than 20 minutes as part of the standard intervention protocol (ie, up to 2 h and 40 min of therapist contact per participant). Similarly, other digital BA interventions have required a trained therapist to review completed assignments and provide personalized feedback for each participant [24,25].

Although these digital interventions require less therapist time than conventional psychotherapy, the ability to deliver digital BA interventions at scale would be increased if interventions did not require human interaction (ie, if they were fully automated). As of now, the existing digital BA protocols investigated in the literature would not effectively assist individuals living in mental health professional shortage areas as there is still a reliance on trained mental health professionals to deliver these interventions, restricting access to services. To our knowledge, a fully automated digital BA protocol for depression has not been examined previously. Furthermore, and in keeping with recent calls for brief and low-intensity mental health interventions in the context of rising mental health issues due to the COVID-19 pandemic [26], the current digital BA intervention focused specifically on the core mechanism of change in BA: increasing enjoyable experiences. Thus, the primary goal of this study was to examine if a fully automated digital intervention designed to promote and increase positive experiences among individuals with elevated depression would result in significant reductions in depressive symptoms over 1 month. The second goal of this study was to assess the impact of the BA digital interventions on symptoms of anhedonia—a symptom of depression that is directly related to positive experiences [6,7,22].

Participants with higher levels of depression (ie, moderate or higher depressive symptoms based on self-report on the 8-item Patient Health Questionnaire [PHQ-8]) were randomized to an automated digital (ie, SMS text message based) intervention prompting individuals to complete daily enjoyable activities for 1 month. We compared this digital BA intervention to a similar automated digital (ie, SMS text message based) intervention that prompted participants to complete healthy living activities (eg, bathing, brushing teeth). Finally, we included a passive control condition that did not prompt any behavior changes. Based on past meta-analytic studies on the effectiveness of both traditional and digital BA treatments, we predict that participants in the enjoyable activities (ie, BA) condition would experience greater decreases in depressive symptoms and anhedonia compared to participants in the healthy activities and passive control conditions. As an exploratory aim, we also investigated if these interventions significantly influenced changes in anxiety and stress over the 4 weeks.

## Methods

### Ethical Considerations

The project was approved by the Florida State University Institutional Review Board (00003451) and was retrospectively registered as a randomized clinical trial (NCT06492824). Participants provided informed consent before participating and were compensated for their time (US $20 for completing the pre- and postsurveys, and an additional US $30 if participants completed at least 90% of their daily checklists over the 4-week time frame, if applicable).

### Participants

Participants were recruited online from the United States via Facebook advertisements from November 2022 to February 2023 (advertisements included in Multimedia Appendix 1). Randomization was conducted during the initial baseline survey using Qualtrics (version XM) software and coding. More specifically, if the interested participants passed the eligibility criteria (discussed in more detail below), the survey was coded to randomize them to either the BA, healthy activity, or passive control conditions.

The inclusion criteria for this study were being 18 years or older, having a current PHQ-8 total score ≥10 (ie, moderate depressive symptoms or greater), and successfully signing up for the SlickText messaging service (ie, providing phone number and following prompts sent to that number to enroll in the service). More specifically, interested participants were first asked to consent to all study procedures and complete the PHQ-8 during the baseline survey. Interested participants who either did not consent to the study or scored lower than a 10 on the PHQ-8, which resulted in the survey terminating, would not be randomized to an experimental condition.

### Measures

#### 8-Item Patient Health Questionnaire

The PHQ-8 is an 8-item measure of current depressive symptoms that assesses 8 of the 9 core symptoms that are the criteria for depression, excluding symptoms of suicidal ideations and behaviors [27]. The questionnaire was administered only during the baseline survey and assessed depressive symptoms over the last 2 weeks to assess eligibility for this study. Each item is scored on a 4-point scale (ranging from 0 to 3; 0=not at all, 1=several days, 2=more than half the days, and 3=nearly every day). Therefore, PHQ-8 total scores range from 0 to 24, and a score ≥10 is considered a validated threshold for clinical levels of depression [27], which is why this measure was used as a screening tool for this study. In this sample, the PHQ-8 total score demonstrated good internal consistency at the baseline assessment (Cronbach α=0.78).

#### 21-Item Depression, Anxiety, and Stress Scale

The 21-item Depression, Anxiety, and Stress Scale (DASS-21) comprises 21 total items; 7 items that assess depressive symptoms, 7 items that assess anxiety symptoms, and 7 items that assess stress symptoms over the past week. Each item is scored on a 4-point scale (ranging from 0 to 3; 0=did not apply to me at all, 1=applied to me to some degree or some of the time, 2=applied to me to a considerable degree or a substantial part of the time, and 3=applied to me very much or most of the time). Participants in this study completed the DASS-21 before and after the 4-week intervention or control period. Past research suggests that the DASS-21 accurately distinguishes features of depression, physical arousal, and psychological tension and agitation. Additionally, these psychometric studies observe acceptable-to-excellent internal consistency and concurrent validity [28]. In this sample, Cronbach α showed good-to-excellent internal consistencies at both the pre- and postassessment depression scores (pre=0.90; post=0.89), anxiety scores (pre=0.88; post=0.86), and stress scores (pre=0.87; post=0.86).

#### Personality Inventory for *Diagnostic and Statistical Manual of Mental Disorders* (Fifth Edition): Anhedonia Subscale

The anhedonia subscale of the Personality Inventory for *Diagnostic and Statistical Manual of Mental Disorders* (Fifth Edition; PID-5) consists of eight items rated on a 4-point Likert scale. Participants in this study completed the PID-5 subscale before and after the 4 weeks, reporting symptoms from over the past 2 weeks. Past research involving this subscale of the PID-5 suggests good internal consistency (Cronbach α ranging from 0.87 to 0.89) in various adult samples [29,30]. In line with these past studies, the PID-5 anhedonia subscale scores in this sample demonstrated good internal consistency according to Cronbach α at the pre- and postintervention assessments (0.75 and 0.85, respectively).

### Procedures

#### Enjoyable Activities, Healthy Activities, and Passive Control Conditions

The active interventional conditions (ie, enjoyable activities [BA] and healthy activities) were delivered automatically via daily SMS text messages using the service SlickText. SlickText provides an SMS text message product that enabled participants to receive automated communications from the research group. Participants randomized to the enjoyable activities and healthy activities conditions were instructed to enroll in SlickText after consenting to this study and provided their phone number, email address, and initials; no other personal information was collected/shared with SlickText.

Participants in the enjoyable activities condition watched an introduction video (approximately 3 minutes in length) that discussed the rationale for the enjoyable activities intervention in combating depressive symptoms during the initial online assessment (video script provided in Multimedia Appendix 2). Additionally, during the initial assessment, enjoyable activities participants were asked to choose 5 enjoyable activities from a large list of various activities from several domains such as social, soothing/fun, physical, and religious (full list provided in Multimedia Appendix 3) that they wanted to do more frequently over the next 4 weeks. Some examples of enjoyable activities from the list were go for a walk, stargaze, go for a drive, go to a service, do some gardening, play with your pet, and text or call one of your friends. Over the next 4 weeks, daily SMS text message reminders were sent, via SlickText, each morning. These SMS text messages reminded participants of the 5 enjoyable activities that they wanted to do more of and encouraged them to complete at least 2 of the enjoyable activities that day. Participants were asked to try to complete at least 2 out of their 5 selected activities each day, though they were told that they could complete more if they wanted. Additionally, participants received a link to complete a daily checklist (ie, short survey) where they reported if they had completed any of their 5 activities on the previous day (example SMS text messages in Multimedia Appendix 4). Next, participants were asked to rate how much they enjoyed doing their completed activities (0‐10 rating) from the previous day on the checklist (short survey). Participants were also asked to complete short weekly questionnaires asking about their mood for the preceding week. At the end of the 4 weeks, enjoyable activities participants completed a postintervention online survey consisting of the same measures from the preintervention survey.

Participants in the healthy activities condition completed most of the same tasks as those participants in the enjoyable activities condition: they completed an initial online preintervention survey and chose 5 enjoyable activities from the same list. However, healthy activities participants were not explicitly asked to complete these activities for their intervention. Instead, participants in the healthy activities condition were encouraged to complete the following 5 activities each day: drinking more water, going to bed early, brushing their teeth, showering or bathing that day, and eating well-balanced meals. Healthy activities condition participants also watched an introduction video (approximately 3 minutes in length) that discussed the rationale for the healthy activities intervention in combating depressive symptoms during the initial online assessment (video script provided in Multimedia Appendix 5).

Daily SMS text reminders were sent, via SlickText, regarding these 5 healthy living activities each morning. In that same message, healthy activities participants received a link to a short online checklist (survey) where they reported which healthy living activities they completed the previous day and how much they enjoyed completing those healthy activities. Consistent with the enjoyable activities condition, healthy activities participants were asked to try to complete at least 2 of the 5 healthy activities each day but could do more if they desired. They were also asked to complete a short weekly questionnaire asking about their mood during the preceding week. At the end of the month, they completed an online postintervention survey consisting of the same measures from the preintervention assessment. Participants in this condition were given the option to complete the enjoyable activities digital intervention after completing the healthy activities condition if they desired.

Control participants completed an initial online survey that also included an introduction video (approximately 1 and a half minutes long; video script provided in Multimedia Appendix 6) explaining the rationale for this specific condition and that researchers wanted to understand the naturalistic course of mood and behavior over 1 month. However, passive control participants were not explicitly asked to complete any enjoyable activities or healthy living activities during the subsequent 4 weeks. Passive control participants were not sent daily text reminders or checklists to complete over the 4 weeks. They were asked to complete the same short weekly questionnaire asking about their mood during the preceding week. At the end of the month, they also completed a postintervention online survey consisting of the same measures from the initial survey. Participants in this condition also had the option to complete the enjoyable activities digital intervention after completing the passive control condition if they desired.

#### Statistical Analyses

All data analyses were performed using SPSS Statistics software (version 27.0; IBM Corp). First, demographic and clinical data were analyzed between the enjoyable activities, healthy activities, and passive control groups using 1-way ANOVAs, independent samples *t* tests, and *χ*^2^ tests. Next, a total of 4 univariate ANOVA models were conducted to determine the effect of group status on changes in symptoms of depression, anhedonia, anxiety, and stress over 4 weeks. Specifically, the experimental condition (ie, enjoyable activities [BA], healthy activities, and passive control) was used as a fixed factor predicting postintervention symptoms; the respective preintervention symptoms were used as covariates in each ANOVA model. Bonferroni corrections were used for all post hoc procedures in the ANOVA models. Considering that multiple models are being conducted, the criterion of statistical significance for all analyses was adjusted and set at .013 (ie, .05 divided by 4). Lastly, demographic and clinical data were analyzed between participants who completed the treatment procedures entirely (ie, returned for follow-up and engaged with their respective interventions according to the completion of daily checklists) and participants who did not complete the treatment procedures or return for the follow-up using independent samples *t* tests and *χ*^2^ tests to assess attrition bias.

#### Power Analysis

The G*Power (Version 3.1; Erdfelder, Faul, and Buchner) application was used to conduct the a priori power analysis [31]. Based on meta-analytic findings, the estimated effect size between BA treatment effectiveness on depression compared to controls that did not receive treatment is expected to be large (Hedges *g*=0.83) [19]. Using 80% power and a large estimated effect size (*f*=0.40 or η^2^=0.14), the recommended total sample size for a 3-group analysis of covariance is 64 participants, and therefore the current sample size is adequately powered to detect the presence of the BA treatment effect on depressive symptoms.

## Results

**Figure 1.**
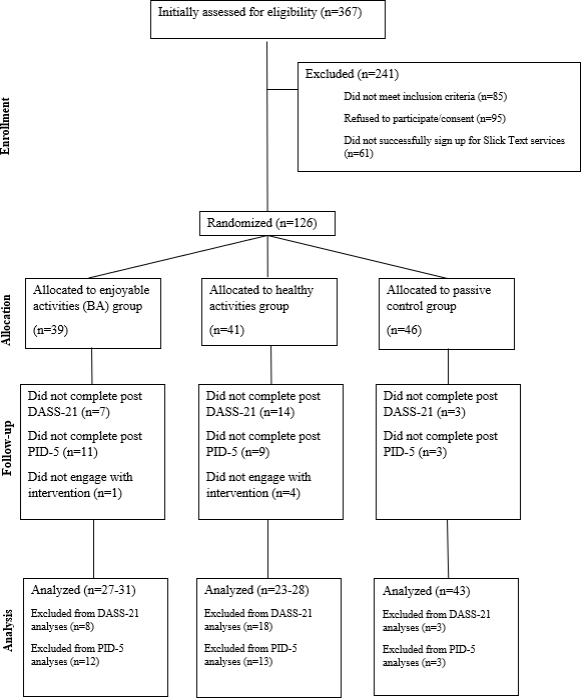
CONSORT (Consolidated Standards of Reporting Trials) diagram. BA: behavioral activation; DASS-21: 21-item Depression, Anxiety and Stress Scale; PID-5: Personality Inventory for *Diagnostic and Statistical Manual of Mental Disorders* (Fifth Edition).

The full sample initially recruited and randomly allocated to the experimental conditions comprised 126 eligible participants: 39 randomized to the enjoyable activities condition, 41 randomized to the healthy activities condition, and 46 randomized to the control condition. Of these 126 randomized participants, 1 participant did not engage with the BA intervention, and 4 participants did not engage with the healthy activities intervention over the month as evidenced by the average number of daily completed activities recorded in their checklists (mean activities 0.28, SD 0.29). Figure 1 displays the CONSORT (Consolidated Standards of Reporting Trials) diagram, which further details the number of participants during initial engagement and recruitment, allocation to the experimental conditions, follow-up data collected, and final analyses for this study.

A total of 97 participants (enjoyable activities: n=31; healthy activities: n=23; passive control: n=43) completed pre- and postintervention measures of depressive, anxiety, and stress symptoms (ie, DASS-21), and 98 participants (enjoyable activities: n=27; healthy activities: n=28; passive control: n=43) completed pre- and postintervention measures of anhedonia (ie, PID-5 anhedonia subscale). The average age of the total sample was 32.20 (SD 7.14) years, and 28% (n=28) of the sample identified as female. In terms of race, half (n=49, 50%) of the participants identified as White, whereas 41% (n=40) of participants identified as African American or Black, 4% (n=3) identified as Asian, 3% (n=3) identified as American Indian or Alaskan Native, 1% (n=1) identified as Pacific Islander, and 1% (n=1) of participants identified as another race. Furthermore, 17% (n=17) identified as Hispanic. Additionally, 34% (n=33) of the sample reported currently taking psychotropic medications (eg, selective serotonin reuptake inhibitor) for symptoms of depression. Table 1 has more detailed information regarding this information as a function of the three conditions.

There were no significant age, gender, or racial composition differences between the enjoyable activities, healthy activities, and passive control participants in this study (all *P* values >.07). Moreover, there were no significant differences in preintervention symptoms of depression (PHQ-8 or DASS-21), anxiety, stress, or anhedonia between the three groups (all *P* values >.33). On average, participants in this study reported moderate-to-severe levels of depressive symptoms at the initial assessment according to both the PHQ-8 and DASS-21. Additionally, there were no differences in current psychotropic medication status between the groups (*P*=.12). There was a significant difference in the average number of daily activities completed (*P*=.002) between the two active intervention conditions such that participants in the healthy activity group completed more activities each day compared to individuals in the enjoyable activities condition. Surprisingly, there were no differences in average enjoyment ratings of these completed daily activities (*P*=.59) between participants in the enjoyable activities and healthy activities conditions; on average, participants in these conditions rated their completed activities as being highly enjoyable (approximately 7 on a 0-10 scale).

**Figure 2.**
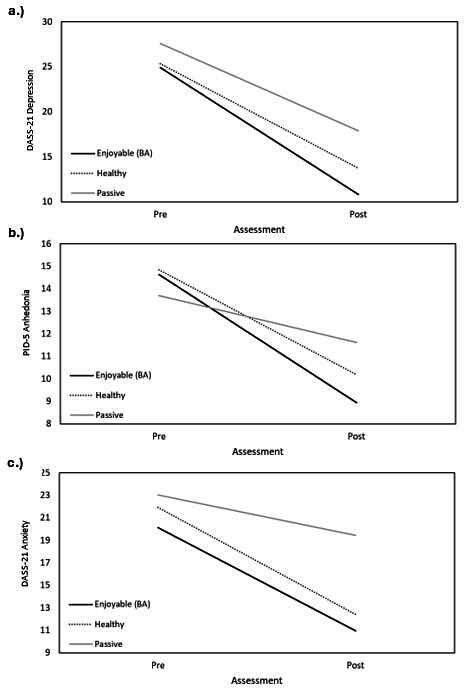
Changes in (A) depressive symptoms, (B) anhedonic symptoms, and (C) anxiety symptoms over 4 weeks by condition. BA: behavioral activation; DASS-21: 21-item Depression, Anxiety, and Stress Scale; PID-5: Personality Inventory for *Diagnostic and Statistical Manual of Mental Disorders* (Fifth Edition).

To assess the impact of the intervention on changes in depressive symptoms, experimental condition (ie, enjoyable activities [BA], healthy activities, and passive control) was entered as a fixed factor in a univariate ANOVA predicting the post–DASS-21 depression score; the pre–DASS-21 depression score was also used as a covariate in the model. The overall ANOVA model was significant (*F*_3,98_=3.06; *P*=.03). Experimental condition emerged as a significant predictor of postintervention depressive symptoms (*F*_2,98_=4.53; *P*=.01; η_p_^2^=0.09). Further, Bonferroni follow-up comparisons revealed that individuals in the BA condition had significantly lower depressive scores after 1 month than individuals in the passive control condition (mean difference −7.27, SE 2.42, 95% CI –13.18 to –1.36; *P*=.01). There were no significant differences in depressive scores for individuals in the BA condition compared to individuals in the healthy activity condition (mean difference −3.55, SE 2.78, 95% CI −10.33 to 3.22; *P*=.61). There was also no difference in postintervention depressive symptoms between individuals in the healthy activity condition and the passive control condition (mean difference −3.72, SE 2.62, 95% CI −10.09 to 2.66; *P*=.48). Lastly, preintervention DASS-21 depressive scores were not significantly associated with postinterevention DASS-21 depressive scores in the model (*F*_1,98_=0.50; *P*=.48; η_p_^2^=0.01). Figure 2A displays changes in depressive symptoms from pre- to postintervention in all three groups.

To further examine the impact of the intervention specifically on symptoms of anhedonia, experimental condition (ie, enjoyable activities [BA], healthy activities, and passive control) was entered as a fixed factor in a univariate ANOVA predicting postintervention PID-5 anhedonia scores; preintervention PID-5 anhedonia scores were also used as a covariate in the model. The overall ANOVA model was not significant (*F*_3,98_=1.87; *P*=.14). Experimental condition also did not significantly predict postintervention anhedonia symptoms (*F*_2,98_=2.52; *P*=.09; η_p_^2^=0.05). Preintervention PID-5 anhedonia scores were not significantly associated with postintervention PID-5 anhedonia scores in the model (*F*_1,98_=0.22; *P*=.64; η_p_^2^=0.00). Figure 2B displays changes in anhedonia symptoms from pre- to postintervention in all three groups.

**Table 1.** Demographic and clinical measures in enjoyable activities, healthy activities, and passive control groups.

			Enjoyable activities (n=31)	Healthy activities (n=28)	Passive control (n=43)	*P* value
**Demographics and intervention**						
	Age (years), mean (SD)		32.48 (7.41)	32.53 (7.63)	31.74 (6.70)	.87
	Gender (female), n (%)		10 (32)	11 (39)	7 (16)	.06
	**Race, n (%)**					.59
		White	12 (39)	15 (54)	22 (51)	
		Black/African American	14 (45)	11 (39)	15 (35)	
		Asian	0 (0)	1 (4)	2 (5)	
	Current psychotropic medication, n (%)		15 (48)	9 (31)	11 (26)	.12
	Number of completed daily activities, mean (SD)		2.86 (0.78)	3.50 (0.77)	—^a^	.002
	Enjoyment rating of activities (0‐10), mean (SD)		7.40 (1.73)	7.18 (1.56)	—	.59
**Clinical, mean (SD)**						
	Pre–PHQ-8^b^		16.81 (3.98)	17.18 (3.58)	15.88 (4.05)	.33
	Pre–DASS-21^c^ depression		24.90 (10.00)	25.33 (12.74)	27.58 (9.07)	.49
	Pre–DASS-21 anxiety		20.13 (10.18)	21.92 (12.41)	23.03 (9.88)	.51
	Pre–DASS-21 stress		25.61 (9.56)	23.86 (11.25)	26.34 (8.35)	.59
	Pre–PID-5^d^ anhedonia		14.63 (5.26)	14.86 (2.98)	13.70 (3.72)	.45
	Post–DASS-21 depression		10.84 (10.52)	13.71 (10.53)	17.91 (9.53)	.01
	Post–DASS-21 anxiety		10.97 (10.17)	12.41 (8.59)	19.44 (8.97)	<.001
	Post–DASS-21 stress		13.87 (10.97)	15.94 (8.98)	17.01 (10.52)	.43
	Post–PID-5 anhedonia		8.94 (5.55)	10.19 (5.19)	11.61 (4.21)	.07

a Not applicable.

b PHQ-8: 8-item Patient Health Questionnaire.

c DASS-21: 21-item Depression, Anxiety, and Stress Scale.

d PID-5: Personality Inventory for *Diagnostic and Statistical Manual of Mental Disorders* (Fifth Edition).

A similar ANOVA model was conducted to examine if either active condition modulated changes in anxiety symptoms. Experimental condition (ie, enjoyable activities [BA], healthy activities, and passive control) was entered as a fixed factor in a univariate ANOVA predicting the postintervention DASS-21 anxiety score; the preintervention DASS-21 anxiety score was also used as a covariate in the model. The overall ANOVA model was significant (*F*_3,98_=5.48; *P*=.002). Experimental condition emerged as a significant predictor of postintervention anxiety symptoms (*F*_2,98_=8.18; *P*=.001; η_p_^2^=0.15). Bonferroni follow-up comparisons revealed that individuals in the BA condition had significantly lower anxiety scores after 1 month than individuals in the passive control condition (mean difference −8.51, SE 2.20, 95% CI −13.88 to −3.14; *P*=.001). There were no significant differences in postintervention anxiety scores between individuals in the BA condition compared to individuals in the healthy activity condition (mean difference −2.35, SE 2.53, 95% CI −3.82 to 8.51; *P*>.99). Additionally, individuals in the healthy activity condition reported significantly lower postintervention anxiety symptoms than individuals in the passive control condition (mean difference −6.17, SE 2.37, 95% CI −11.94 to −0.39; *P*=.03). Lastly, preintervention DASS-21 anxiety scores were not significantly associated with postintervention DASS-21 anxiety scores in the model (*F*_1,98_=0.02; *P*=.88; η_p_^2^=0.00). Figure 2C displays changes in anxiety symptoms from pre- to postintervention in all three groups.

A final ANOVA model was conducted to examine if participants in either of the active conditions experienced greater changes in symptoms related to stress. Consistent with the previous ANOVA models, experimental condition (ie, enjoyable activities [BA], healthy activities, and passive control) was entered as a fixed factor in a univariate ANOVA predicting postintervention DASS-21 stress scores; preintervention DASS-21 stress scores were also used as a covariate in the model. The overall ANOVA model was not significant (*F*_3,98_=0.70; *P*=.55). Experimental condition also did not significantly predict postintervention stress symptoms (*F*_2,98_=0.92; *P*=.40; η_p_^2^=0.02). Lastly, preintervention DASS-21 stress scores were not significantly associated with postintervention DASS-21 stress scores in the model (*F*_1,98_=0.26; *P*=.61; η_p_^2^=0.00).

There were no significant age, gender, or racial composition differences between participants who completed the intervention procedures and participants who did not complete the study (all *P* values >.05). Additionally, there were no significant differences in preintervention symptoms of depression, anxiety, stress, or anhedonia between the two groups (all *P* values >.06). Lastly, there were no differences in current psychotropic medication status between the groups (*P*=.14).

## Discussion

This study examined the efficacy of a fully automated digital BA intervention at reducing depressive symptoms and anhedonia over 1 month in individuals with moderate or greater current symptoms of depression. Compared to a fully automated digital active control condition in which individuals were prompted to increase healthy activities and a control condition in which individuals were not prompted to make any lifestyle changes, individuals who completed the fully automated digital BA intervention experienced greater reductions in their depressive symptoms compared to the control condition; there were no significant differences in depressive symptoms changes between the healthy activities and control condition. However, there did not appear to be specific reductions in anhedonia related to the digital BA intervention. Moreover, individuals that completed either the active digital intervention (ie, enjoyable activities [BA] or healthy activities) also reported significant decreases in symptoms of anxiety over the 4 weeks compared to the passive control condition. Neither digital intervention appeared to be effective at reducing symptoms attributed to stress.

These findings are directly in line with past research that has demonstrated BA as a frontline treatment for depressive disorders [18,19] and further supports the digital implementations of BA treatment for reducing symptoms of depression [22]. Unlike past digital BA projects, this study implemented a completely automated treatment protocol requiring no human intervention. Considering the rising prevalence rates of depression compounded by limited access to evidence-based mental health services and experts, there is an imperative need to create scalable effective mental health interventions that can be widely disseminated [26].

Individuals who completed the digital BA intervention did not experience a significant decrease in symptoms of anhedonia compared to individuals assigned to the other two experimental conditions. Anhedonia is a particularly deleterious symptom of depression in that it is associated with negative outcomes such as a prolonged disease course, worse long-term prognosis, and higher suicide rates, making it a difficult symptom to modulate [32,33]. The current results are at odds with other studies that have observed that traditional (ie, nondigital) BA treatment can alleviate this particular core symptom of depression [34] and neuroimaging studies that further suggest that successful BA leads to increases in brain systems associated with reward processes, such as increases in activation of the dorsal striatum linked to reward anticipation, which is typically reduced in individuals experiencing heightened depression and anhedonia [35,36]. Although the current digital BA intervention did not specifically reduce symptoms of anhedonia, it is still plausible that other digital versions of BA treatment could likely incite the same mechanism of change seen in traditional BA in that the intervention successfully increases positive more rewarding experiences, which in turn decreases negative affect and perhaps more specifically anhedonia.

Participants who completed both the BA and healthy activities interventions also experienced significant decreases in anxiety symptoms over 1 month. Despite both conditions prompting daily lifestyle changes, reductions in anxiety symptoms seen in the BA and healthy activities conditions appear to be independent of one another. Past research has demonstrated that BA treatment can be effective at treating symptoms of anxiety [37,38], which most researchers attribute to disruptions of avoidant behaviors and behavioral inhibition, which are core features of anxiety [37]. Regarding healthy activities such as eating more well-balanced meals, bathing regularly, and going to bed early, past research has demonstrated that individuals with elevated anxiety are more likely to report disruptions of these psychosocial rhythms and poor general health even after controlling for current depressive symptoms [39]. Factors such as poor nutrition and impaired sleep share a bidirectional relationship with elevated symptoms of anxiety [40,41]. Therefore, promoting more consistent participation in these healthy activities daily should affect anxiety symptoms. Thus, it is possible that BA and healthy activities reduced anxiety symptoms via different mechanisms of change—though this possibility requires further evidence.

Surprisingly, there were no significant differences in the average enjoyment ratings of completed activities between the BA (ie, enjoyable activities) and healthy activities conditions. Moreover, individuals in the healthy activity condition completed more activities over the month compared to individuals in the BA condition. It is possible that although participants reported similar levels of enjoyment on average in their completed activities, there are likely other key variables that were not assessed in this study regarding these experiences that significantly alleviated low mood. For example, it is plausible that the amount of time spent doing the activity influences its effectiveness on positive affect and therefore negative affect. Additionally, it is possible that completing activities from different domains might be more effective at increasing positive affect (eg, social activities compared to individual activities, physical activities compared to more sedentary hobbies). Future studies should expand on the activity information that is gathered from participants during BA interventions to further elucidate the mechanisms underlying reductions in depressive symptoms seen in BA treatment.

Lastly, we observed that almost half of the sample identified as Black or African American in this randomized controlled trial. Although this number is disproportionate to the percentage of Black or African Americans in the United States in general, this percentage might be more proportionate when considering differences in ethnic and racial prevalence rates of depression in the United States. Studies posit that rates of depression are higher for individuals identifying as Black or African American [42-44], which might be reflected here considering recruitment for the study was aimed toward individuals with low mood. Relatedly, it is commonly observed that Black or African American individuals seek traditional mental health help at much lower rates than White Americans [45]. It is plausible that the unique virtual and automated design of the present intervention was more attractive for individuals as it was more convenient and posed fewer barriers compared to other interventions with in-person aspects.

This study has limitations worth noting. Participants in this study were not administered clinical interviews to confirm the diagnosis of a depressive disorder. Although higher PHQ-8 scores, specifically total scores ≥10, suggest an increased likelihood of a depressive disorder [46], this was not confirmed by clinical interview, so the clinical status of our participants is unknown. More detailed information regarding depressive disorders is typically collected in diagnostic interviews, such as number of past episodes, age of onset, and the duration of the current episode; these are all crucial factors that have been associated with worse treatment response [47] that could be examined in future studies. Relatedly, overall depressive symptoms improved for participants in each condition over 1 month in this study. This is likely accounted for by regression toward the mean. More specifically, it is possible that depressive symptoms reported at the initial session reflected extreme symptoms and would therefore be a motivating factor to participate in this study. Therefore, reassessing these symptoms again a month later could more naturally reflect their average depressive ratings regardless of experimental condition. Additionally, a positive placebo response is often seen in clinical trials for depression [48]. However, despite these possibilities, there is still evidence to suggest that the BA intervention particularly led to more significant decreases in depression compared to the passive control group. Additionally, it would have been important to understand how other comorbid mental health disorders, such as anxiety disorders or substance use disorders, might have influenced responsiveness to the current digital interventions. Although current psychotropic medication information was collected from participants in the study and examined between the three experimental conditions, we did not assess for detailed information regarding current psychotherapy, which limits our findings. It is plausible that there might have been differences between the three groups regarding current therapy enrollment outside of the current interventions, which could have influenced the effectiveness of the digital BA intervention.

The current randomized controlled trial was not preregistered and used Facebook to recruit participants. Although Facebook allowed the research team to conveniently reach a large number of interested diverse participants from around the United States in a short amount of time, the overall quality of these participants could not be established. However, the sound psychometric properties (ie, Cronbach α ranging from 0.75 to 0.90) of the various clinical measures used as outcomes suggest the validity and reliability of data collected from the sample. Future studies might replicate these results using other online platforms such as Amazon Mechanical Turk, which allows researchers to recruit from more “vetted” registered participant pools [49]. This study also had significant strengths that are worth noting. As discussed earlier, this study is the first to implement a fully automated digital BA treatment for depression that requires no human intervention, which is highly scalable and could be widely accessible. Additionally, this study did not predominately involve White participants, which is a chronic issue in psychological research that typically hinders the generalizability of results [50].

In summary, this study demonstrated the efficacy of a fully automated BA intervention (ie, promoting daily enjoyable activities) for decreasing depressive symptoms over 1 month. Additionally, both automated digital intervention protocols (ie, BA and healthy activities) significantly reduced symptoms of anxiety over 4 weeks. Neither digital intervention had a significant effect on anhedonia or stress symptoms.

## Supplementary material

10.2196/54252 Multimedia Appendix 1 Advertisements.

10.2196/54252 Multimedia Appendix 2 Enjoyable activities video script.

10.2196/54252 Multimedia Appendix 3 List of enjoyable activities.

10.2196/54252 Multimedia Appendix 4 Example of SMS text messages.

10.2196/54252 Multimedia Appendix 5 Healthy activities video script.

10.2196/54252 Multimedia Appendix 6 Passive condition video script.

10.2196/54252 Checklist 1 CONSORT-EHEALTH checklist (V 1.6.1)

## Abbreviations

BA: behavioral activation
CBT: cognitive behavioral therapy
CONSORT: Consolidated Standards of Reporting Trials
DASS-21: 21-item Depression, Anxiety, and Stress Scale
PHQ-8: 8-item Patient Health Questionnaire
PID-5: Personality Inventory for *Diagnostic and Statistical Manual of Mental Disorders* (Fifth Edition)
