# Supplementary material for: Examining a Fully Automated Mobile-Based Behavioral Activation Intervention in Depression: Randomized Controlled Trial
Source: JMIR Ment Health. 2024 Aug 30;11:e54252. doi: 10.2196/54252 (PMC11378696; doi:10.2196/54252)
Supplement: Multimedia Appendix 1 [file mental-v11-e54252-s001.docx]

The RAD lab at Florida State University is looking for your participation in our research study examining the impact of a one-month long intervention for depressive symptoms.

We are conducting a fully online study examining the text message-based interventions that promote healthy and enjoyable activities.

We are looking for interested people who are currently experiencing any depressed, sad, or down mood that might be impairing or distressing.

This study is completely digital (online surveys and texting), and you will be paid $40 plus an additional $30 in bonuses depending on your level of participation in the project. Payment will be with a digital Amazon gift card.

What to expect in the study:

1. Online surveys (e.g., short daily checklists and short weekly surveys).

2. Daily text reminders to complete activities.

Interested in participating? Fill out the survey below:

<https://fsu.qualtrics.com/jfe/form/SV_5jN4k9eBBlx2XtA>

Want more information?

Please contact us at the RAD Lab!

Call: 850-320-7087

Email: hajcakip@gmail.com

For more information about our current research, please visit our website!

[https://cpr-lab.weebly.com/](https://cpr-lab.weebly.com/?fbclid=IwAR3G6-W7srDAJyKCqyaOZkv6YKyufb7LemlNEo_wI47kyi7qvXvL503TNC8)

The RAD lab at Florida State University is looking for your participation in our research study examining the course of depressive symptoms over one month.

We are looking for interested people who are currently experiencing any depressed, sad, or down mood that might be impairing or distressing.

This study is completely digital (online surveys), and you will be paid $40 for your participation. Payment will be with a digital Amazon gift card.

What to expect in the study:

1. Online surveys (e.g., short weekly surveys).

Interested in participating? Fill out the survey below:

https://fsu.qualtrics.com/jfe/form/SV_5cYgbbVBnJwTpZA

Want more information?

Please contact us at the RAD Lab!

Call: 850-320-7087

Email: hajcakip@gmail.com

For more information about our current research, please visit our website!

[https://cpr-lab.weebly.com/](https://cpr-lab.weebly.com/?fbclid=IwAR3G6-W7srDAJyKCqyaOZkv6YKyufb7LemlNEo_wI47kyi7qvXvL503TNC8)
