# Supplementary material for: Examining a Fully Automated Mobile-Based Behavioral Activation Intervention in Depression: Randomized Controlled Trial
Source: JMIR Ment Health. 2024 Aug 30;11:e54252. doi: 10.2196/54252 (PMC11378696; doi:10.2196/54252)
Supplement: Multimedia Appendix 2 [file mental-v11-e54252-s002.docx]

**BA Digital Intervention Video Script:**

“Hello, my name is Nick Santopetro and I am a graduate student studying clinical psychology at Florida State University.”

“First, I want to thank you for showing interest in our study and agreeing to be a part of it!”

“What I want to do now is briefly go over what we will be asking you to complete in this study over the next four weeks and the rationale for why we are asking you to complete these tasks.”

“In short, we will be encouraging you via text message to complete enjoyable activities on a daily basis over the next month. We will be encouraging you to engage in specific activities that you find enjoyable. On the following page you will be choosing a total of five activities from a large list of activities. So, think about things that you like doing, and that you might like to do more. Our goal is for you to complete at least two out of theses five activities on a daily basis. Again, you will have the freedom to choose which of your five activities you want to complete that day as long as you are attempting to do at least two of them (you are more than welcome to do more than two each day). Over the next month, we will send a very brief checklist via text message every day, and that checklist will ask you 1.) what activities you completed the day before, and 2.) how much you enjoyed doing those completed activities.”

“The design of this present study is based on a treatment called Behavioral Activation which is predominately utilized to treat symptoms of depression. According to this approach, the key to reducing symptoms of depression is to develop healthier patterns of behavior – to increase important and/or enjoyable activities that help you feel fulfilled and give life more purpose and fun. This is important because when you accomplish activities that are closely linked to what you truly value and enjoy in life, you are more likely to have positive and enjoyable experiences—and those experiences will improve how you feel and think about your life. It is difficult to feel depressed and hopeless if you are regularly doing activities that you feel are valuable and worthwhile and that bring you a sense of pleasure and accomplishment.

Many individuals experiencing depressive symptoms feel tired and lack the motivation to do enjoyable activities; it is typical for people to think that they should wait for the motivation to come before they do enjoyable activities. Behavioral activation takes the opposite approach: the idea is to change behavior first, with the idea that motivation will increase later. Here is an example: lots of people struggle with motivation to exercise; yet, almost everyone feels good *after* they exercise. If you wait for motivation, you might not exercise; but if you just go exercise, you’ll feel more motivated to do it next time, and you’ll feel better. Over the next month, we’re just going to send you reminders to do more of the things that you find enjoyable—the hope is that it’ll increase the frequency and time you spend doing things that make you feel good ”

“If you have any questions or concerns about the study at any time over the next four weeks please do not hesitate to send an email to hajcakip@gmail.com or give us a call at 850-320-7087.”

“Thank you again for agreeing to participate in our study!”
