# Supplementary material for: Examining a Fully Automated Mobile-Based Behavioral Activation Intervention in Depression: Randomized Controlled Trial
Source: JMIR Ment Health. 2024 Aug 30;11:e54252. doi: 10.2196/54252 (PMC11378696; doi:10.2196/54252)
Supplement: Multimedia Appendix 3 [file mental-v11-e54252-s003.docx]

Go for a walk

Go for a jog or run

Go for a swim

Go for a hike or walk in nature

Go to a park

Go for a bike ride

Go fishing

Go rock climbing

Get out on the water- go kayaking or canoeing

Stargaze

Watch the sunrise or sunset

Go sightseeing in your own town (try somewhere new)

Go for a drive

Do some photography - take photos around town or in nature

Start a garden or do some gardening

Have a picnic

Sit or lay in the sun

Do yoga

Practice or play a sport (e.g., soccer, golf, karate, volleyball, bowling, etc.)

Weight training at a gym or at home

Go watch a sporting event

Go to a zoo or aquarium

Go shopping at a new store

Buy or wear a new outfit

Buy a new plant, or take care of your plants

Make plans to hang out with friends outside of where you live

Go to dinner with friends/have friends over for dinner

Go on a date

Text/ call one of your friends

Text/ call one of your family members

Do something nice for someone you care about

Spend time with your family

Play with your pet

Start a conversation with someone new

Volunteer or help someone

Make a gift for someone and give it to them

Go to church/ attend a service

Meditate for 30 minutes

Get a manicure or pedicure

Get a massage

Go see live music

Listen to your favorite music

Sing your favorite song

Dance to your favorite song

Learn or practice a musical instrument

Read a book

Read a comic book

Write in a journal

Listen to a lecture on an interesting topic

Listen to an audiobook

Listen to a podcast you like

Listen to a new podcast

Watch one of your favorite movies or TV shows

Watch one of your favorite comedians

Watch something funny on YouTube

Play video games

Work on a cross word or some other puzzle

Draw or paint something new

Craft/make something by hand (e.g., crochet, model building)

Cook for yourself/cook something new

Bake something from scratch

Repair or fix something

Learn a new skill
