# Supplementary material for: Examining a Fully Automated Mobile-Based Behavioral Activation Intervention in Depression: Randomized Controlled Trial
Source: JMIR Ment Health. 2024 Aug 30;11:e54252. doi: 10.2196/54252 (PMC11378696; doi:10.2196/54252)
Supplement: Multimedia Appendix 4 [file mental-v11-e54252-s004.docx]

*Example text message for BA participants:*

“Hello from the RAD Lab! This is your reminder to complete at least 2 out of your 5 selected enjoyable activities today.

Please use the following link to complete your checklist about yesterday's activities: [https://fsu.qualtrics.com/jfe/form/SV_4SZQJaWdLqGkVym?pptIDCode={{first_name}}&cl=1](https://fsu.qualtrics.com/jfe/form/SV_4SZQJaWdLqGkVym?pptIDCode=%7B%7Bfirst_name%7D%7D&cl=1)”

*Example text message for Active control participants:*

“Hello from the RAD Lab! This is your reminder to complete at least 2 out of your 5 healthy activities today.

Please use the following link to complete your checklist about yesterday's activities: [https://fsu.qualtrics.com/jfe/form/SV_e4KlZ8Hk1Tplumy?pptIDCode={{first_name}}&cl=1](https://fsu.qualtrics.com/jfe/form/SV_e4KlZ8Hk1Tplumy?pptIDCode=%7B%7Bfirst_name%7D%7D&cl=1)”
