# Supplementary material for: Examining a Fully Automated Mobile-Based Behavioral Activation Intervention in Depression: Randomized Controlled Trial
Source: JMIR Ment Health. 2024 Aug 30;11:e54252. doi: 10.2196/54252 (PMC11378696; doi:10.2196/54252)
Supplement: Multimedia Appendix 5 [file mental-v11-e54252-s005.docx]

**Healthy Life Digital Intervention Video Script:**

“Hello, my name is Nick Santopetro and I am a graduate student studying clinical psychology at Florida State University.”

“First, I want to thank you for showing interest in our study and agreeing to be a part of it!”

“What I want to do now is briefly go over what we will be asking you to complete in this study over the next four weeks and the rationale for why we are asking you to complete these tasks.”

“In short, we will be encouraging you via text message to complete healthy activities on a daily basis over the next month. We will be encouraging you to engage in healthy activities; specifically, good personal hygiene (like bathing and brushing your teeth), drinking plenty of water, eating balanced meals, and getting a good nights sleep. Our goal is for you to complete at least two out of these five healthy activities on a daily basis. You will have the freedom to choose which of the five healthy activities you want to complete that day as long as you are attempting to do at least two of them (you are more than welcome to do more than two each day). Over the next month, we will send a very brief checklist via text message every day, and that checklist will ask you 1.) what activities you completed the day before, and 2.) how much you enjoyed doing those completed activities.”

“The design of this present study is based on the idea that regular psychosocial rhythms (such as when/what you eat or when you go to bed) are impaired in individuals experiencing depression. For example, an individual experiencing elevated symptoms of depression may have trouble falling asleep at night and does not feel rested the next day; or they may have issues with eating regular nutritious meals. In fact, sleep disturbances and changes in appetite are common symptoms of depressive disorders. “

“Many individuals experiencing depressive symptoms lack the motivation to do these activities; it is typical for people to think that they should wait for the motivation to come before they do healthy activities. This intervention takes the opposite approach: the idea is to change behavior first, with the idea that motivation will increase later. Here is an example: people might struggle with the motivation to bathe—they might not feel like it; yet, almost everyone feels good after they have taken a hot shower or bath. If you wait for motivation, you might not bathe; but if you just bathe, you’ll feel more motivated to do it next time, and you’ll feel better. Over the next month, we’re just going to send you reminders to do more things that are healthy—and the hope is that it’ll increase the frequency and time you spend doing things that are better for you. ”

“If you have any questions or concerns about the study at any time over the next four weeks please do not hesitate to send an email to hajcakip@gmail.com or give us a call at 850-320-7087.”

“Thank you again for agreeing to participate in our study!”
