# Supplementary material for: Examining a Fully Automated Mobile-Based Behavioral Activation Intervention in Depression: Randomized Controlled Trial
Source: JMIR Ment Health. 2024 Aug 30;11:e54252. doi: 10.2196/54252 (PMC11378696; doi:10.2196/54252)
Supplement: Multimedia Appendix 6 [file mental-v11-e54252-s006.docx]

**Passive Control Video Script:**

“Hello, my name is Nick Santopetro and I am a graduate student studying clinical psychology at Florida State University.”

“First, I want to thank you for showing interest in our study and agreeing to be a part of it!”

“What I want to do now is briefly go over what we will be asking you to do in this study over the next four weeks and the rationale for why we are asking you to complete these tasks.”

“In short, we are interested in understanding the naturalistic course of mood and behavior over the course of one month. To understand this, we will be sending you short weekly measures via text message to assess mood for each week. At the end of those four weeks, you will have the chance to participate in a free four-week text-based program that is designed to improve mood...if you choose to do so. Your involvement in the current study simply involves completing weekly measures via text message each week; you will be paid $20 for completing the initial survey today and an additional $20 if you complete the final survey at the end of the month.”

“If you have any questions or concerns about the study at any time over the next four weeks please do not hesitate to send an email to hajcakip@gmail.com or give us a call at 850-320-7087.”

“Thank you again for agreeing to participate in our study!”
